# Defective mitophagy and the etiopathogenesis of Alzheimer’s disease

**DOI:** 10.1186/s40035-022-00305-1

**Published:** 2022-06-03

**Authors:** Kuan Zeng, Xuan Yu, Yacoubou Abdoul Razak Mahaman, Jian-Zhi Wang, Rong Liu, Yi Li, Xiaochuan Wang

**Affiliations:** 1 Department of Psychiatry, Wuhan Mental Health Center, Wuhan, 430012 China; 2 Co-Innovation Center of Neurodegeneration, Nantong University, Nantong, 226001 China; 3 Department of Pathophysiology, School of Basic Medicine, Key Laboratory of Education Ministry/Hubei Province of China for Neurological Disorders, Tongji Medical College, Huazhong University of Science and Technology, Wuhan, 430030 China; 4 Wuhan Hospital for Psychotherapy, Wuhan, 430012 China; 5 Shenzhen Huazhong University of Science and Technology Research Institute, Shenzhen, 518000 China

**Keywords:** Mitophagy, Alzheimer’s disease, PINK1, Tau, Aβ

## Abstract

Accumulation of impaired mitochondria and energy metabolism disorders are non-negligible features of both aging and age-related neurodegeneration, including Alzheimer’s disease (AD). A growing number of studies suggest that mitophagy disorders play an important role in AD occurrence and development. The interaction between mitophagy deficits and Aβ or Tau pathology may form a vicious cycle and cause neuronal damage and death. Elucidating the molecular mechanism of mitophagy and its role in AD may provide insights into the etiology and mechanisms of AD. Defective mitophagy is a potential target for AD prevention and treatment.

## Introduction

Alzheimer’s disease (AD) is a progressive neurodegenerative brain disorder substantially damaging the brain’s structure and function [1]. AD is not only the leading cause of dementia, but also one of the greatest healthcare challenges of the twenty-first century [2]. Today, more than 50 million people suffer from dementia worldwide, with 60%–80% of them caused by AD, and this number is expected to triple by 2050 [3]. The gradual cognitive decline of AD patients is associated with brain atrophy, amyloid plaque deposition, and neurofibrillary tangle formation [4]. Several AD pathogenic mechanisms have been studied, including amyloid beta (Aβ) aggregation and deposition with plaque development, Tau hyperphosphorylation with tangle formation, and neurovascular dysfunction, along with other mechanisms such as cell-cycle abnormalities, inflammatory processes, oxidative stress, and mitochondrial dysfunction [5]. Despite the great efforts to understand its basic biology and clinical pathophysiology, no effective strategies have emerged to prevent and treat AD. Significant investments in drug discovery programs over the past two decades have yielded some important insights but no blockbuster drugs to alter the course of the disease [6]. Thus, much work is still required to explore the pathogenesis of AD and find key proteins and mechanisms that could be targeted for a better treatment.

As early as 1991, Blass and Gibson had already suggested that abnormalities in oxidative metabolism and mitochondria play a crucial role in AD [7]. Since then, hundreds of studies have documented mitochondrial abnormalities in AD and elucidated the potential molecular mechanisms and cellular consequences of mitochondrial defects. A study reported that AD patients have significantly higher mitochondrial DNA, mitochondrial protein, and mitochondria levels in neurons, particularly in large vulnerable neurons of the hippocampus and neocortex, compared to age-matched or young controls [8]. In 2004, Swerdlow and Khan proposed the “mitochondrial cascade hypothesis”, which has inspired many researchers. They pointed out that the mitochondrial dysfunction plays a primary role in the pathology of sporadic late-onset AD (determined by genetic and environmental factors) and drives both Aβ plaque and neurofibrillary tangle formation [9]. With the growing number of researchers interested in autophagy and mitophagy, mitophagy—the process by which cells detect and clear damaged mitochondria—has been found to be partly dysfunctional in AD patients, causing accumulation of dysfunctional mitochondria in neurons. In 2017, Fang and Bohr et al. hypothesized that defective mitophagy is a key risk factor for the initiation and progression of AD [10]. They provided evidence that mitophagy is indeed compromised in AD, including human brains, human stem cells, and different AD models. Meanwhile, inducing mitophagy ameliorates cognitive deficits and AD pathologies [11]. Fang et al. further extended the theories that as an original region of Tau pathology in AD and a pivotal component of memory system, the entorhinal cortex might also be affected by age-related defective mitophagy [12]. Several research groups have studied the implications of defective mitophagy in AD and its interaction with Tau and Aβ pathology, but the conclusions are quite intricate. Thus, this review discusses the mechanisms of mitophagy in AD pathogenesis and its potential in AD prevention and treatment.

### Molecular mechanisms of mitophagy

Autophagy (meaning macroautophagy here) is a process through which double-membrane vesicles, called autophagosomes, deliver organelles and proteins to the lysosome for degradation [13]. Autophagic dysfunction is intimately connected to human physiology and diseases such as cancer, neurodegeneration, microbial infection, and aging [14]. Autophagy not only compensates for nutrient loss by recycling the cellular components, but also exerts quality control by selectively removing defective organelles [15]. In 1966, Christian de Duve and Robert Wattiaux first visualized mitophagy in mammalian cells [16]. In 2005, John J. Lemasters first proposed the term “mitophagy” to designate the autophagy of mitochondria [17]. Mitophagy is a highly conserved cellular process. Its molecular mechanism has been studied and reported in yeast, *Caenorhabditis elegans*, *Drosophila melanogaster*, *Danio rerio*, and mammals [18, 19]. Mitochondria are indispensable for cell metabolism and physiological activities, and mitochondrial damage is associated with various pathological changes [20]. Damaged mitochondria impair the maintenance of cell homeostasis, and thus the cell quality control mechanism able to restore and maintain energy metabolism [21]. Mitophagy is the process of removing dysfunctional or excess mitochondria to adjust their number and maintain a balanced energy metabolism [10]. The mechanisms of mitophagy are divided into ubiquitin-dependent and non-ubiquitin-dependent processes [22]. Many studies have already revealed the intricacies of these interactions in the signaling and execution pathways of mitophagy.

#### Ubiquitin-dependent mitophagy

Ubiquitin-dependent mitophagy mainly refers to the putative kinase 1 (PINK1)-parkin pathway, mainly involving the phosphatase and tensin homologue (PTEN)-induced PINK1 and the E3 ubiquitin ligase parkin. Harper et al. have described this pathway as containing three positively acting elements: a mitochondrial damage sensor (PINK1), a signal amplifier (parkin), and a signal effector (ubiquitin chains) [23]. Together, these proteins determine how the dysfunctional mitochondria will be captured and sequestrated by the autophagy machinery.

PINK1 binds to the translocase of the outer membrane complex through its N-terminal mitochondrial-targeting sequence [24]. Under physiological conditions, PINK1 is transported to the inner mitochondrial membrane, and the mitochondrial-targeting sequence and transmembrane segment of PINK1 reach the translocase of the inner-membrane (TIM) complex [23, 25]. Presenilins-associated rhomboid-like protein, an inner mitochondrial membrane protease, truncates the transmembrane segment of PINK1 and releases a 52-kDa ΔN-PINK1 fragment into the cytoplasm [26]. This ΔN-PINK1 is ubiquitylated by an N-end rule ubiquitin ligase and degraded by the proteasome to maintain low levels of cellular PINK1 [27, 28]. However, mitochondrial damage blocks the transport and processing pathways of PINK1. This results in the aggregation of active PINK1 at the mitochondrial outer membrane after a mitochondrial membrane potential decrease. The auto-phosphorylated, activated PINK1 that accumulates on the outer membrane then activates the E3 ubiquitin ligase parkin through a multi-step positive feedback mechanism [29, 30]. Next, parkin-dependent ubiquitin chains are assembled on the outer membrane, promoting the recruitment of ubiquitin-binding mitochondrial autophagy receptors, which in turn facilitates their capture and sequestration by autophagosomes [18, 31].

**Fig. 1:**
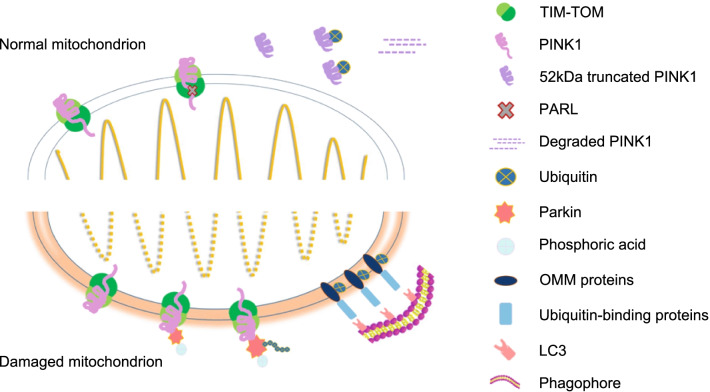
Ubiquitin-dependent mitophagy. PINK1 binds to the TOM complex and, under physiological conditions, is transported to the inner mitochondrial membrane and interacts with the TIM complex. PARL truncates the transmembrane segment of PINK1, releasing the 52-kDa ΔN-PINK1 into the cytoplasm. ΔN-PINK1 is then ubiquitylated and degraded by proteasomes. However, when mitochondria are damaged, PINK1 aggregates on the mitochondrial outer membrane and activates parkin. Parkin-dependent ubiquitin chains are then assembled on the outer membrane. Parkin ubiquitinates several mitochondrial outer membrane proteins, such as VDAC, Mfn2, and DRP1, which are then recognized by the ubiquitin-binding proteins, including OPTN, p62, NDP52, and NBR. These proteins recognize phosphorylated polyubiquitin chains and initiate autophagosome formation by binding to LC3. Finally, the damaged mitochondria recruit the autophagy pathway and fuse with the lysosome, leading to mitochondrial degradation

The activation process of parkin has attracted much attention from mitophagy researchers. In cells with healthy mitochondria, parkin is found in the cytoplasm in an autoinhibited form [32]. However, mitochondrial damage can modify parkin and activate its E3 ubiquitin ligase activity. PINK1 initiates these parkin-activating modifications, which include phosphorylation, multiple conformational changes, and association with Ser65-phosphorylated ubiquitin [23]. In turn, parkin ubiquitinates several mitochondrial outer membrane proteins, such as the voltage-dependent anion-selective channel protein (VDAC), mitofusin 2 (Mfn2), and dynamin-1-like protein (DRP1), which are then recognized by the ubiquitin-binding proteins, including optineurin (OPTN), p62, calcium binding and coiled-coil domain 2 (NDP52), and protein phosphatase 1 regulatory subunit 9A [18, 19, 33–35]. These proteins recognize the phosphorylated polyubiquitin chains and initiate autophagosome formation by binding to microtubule-associated protein 1A/1B-light chain 3 (LC3). Finally, the damaged mitochondria are recruited to the autophagy pathway and fuse with the lysosome, leading to mitochondrial degradation (Fig. 1).

Several other E3 ubiquitin ligases also participate in mitophagy, including glycoprotein 78, SMAD specific E3 ubiquitin protein ligase 1, Seven in absentia homolog 1, mitochondrial ubiquitin ligase activator of NFKB 1, and Ariadne RBR E3 Ub protein ligase 1 [36–40].

#### Non-ubiquitin-dependent mitophagy

Besides participating in ubiquitin-dependent mitophagy, some mitochondrial proteins act as mitophagy receptors and directly target dysfunctional mitochondria towards autophagosomes for degradation [36]. The upstream autophagy regulator AMBRA1 (activating molecule in Beclin-1-regulated autophagy) induces mitophagy by binding to the autophagosome adapter LC3 via its LC3-interacting region (LIR) motif and regulates both the parkin-dependent and the parkin-independent pathways [37]. The mitochondrial outer membrane protein and mitophagy receptor FUN14 domain-containing protein 1 (FUNDC1) initiates mitophagy in mammalian cells by recruiting LC3 through its LIR motif [38]. Furthermore, FUNDC1 regulates mitochondrial dynamics and mitophagy under hypoxic conditions through the mitochondrial fusion/fission proteins DRP1 and optic atrophy 1 (OPA1) [39]. NIP3-like protein X (NIX) is a mitophagy receptor of reticulocytes involved in programmed mitophagy during cell differentiation; it eliminates mitochondria during their maturation [40, 41]. B cell lymphoma 2 (BCL2) interacting protein 3 (BNIP3), an NIX analog and a member of the B cell lymphoma 2 homology domain 3 (BH3)-only subfamily of pro-apoptotic Bcl-2 proteins, is another mitophagy receptor that induces mitophagy in cardiac myocytes [42]. BNIP3 regulates PINK1 stabilization on the mitochondrial outer membrane by inhibiting its proteolysis and promotes PINK1/parkin-mediated mitophagy under physiological conditions [43]. Bcl-2-like protein 13, another member of the Bcl-2 family, induces mitochondrial fragmentation in the absence of Drp1 and induces parkin-independent mitophagy in mammalian cells [44]. FK506-binding protein (FKBP) 8, a member of the FKBP family, recruits LC3A to damaged mitochondria, inducing mitophagy via its N-terminal LIR motif [45]. Specifically, cardiolipin, a phospholipid localized in the inner mitochondrial membrane, supports the function of the respiratory chain in healthy mitochondria. Upon mitochondrial damage, cardiolipin translocates to the outer surface of the outer mitochondrial membrane and interacts with LC3, serving as an “eat-me” signal of mitophagy in neuronal cells [46, 47].

All these receptors are located on the mitochondrial membrane and bind the nascent autophagosome-related proteins via their LIR motif [10, 48]. The LC3 family belongs to the autophagy-related protein 8 (Atg8) family, whose members bind to phosphatidylethanolamine, an integral membrane component of the autophagosome [49, 50]. Another member of the Atg8 family is the gamma-aminobutyric acid receptor-associated protein (GABARAP). They both participate in the mitophagy process as binding partners. LC3 elongates the phagophore membrane and GABARAP helps the membrane envelop the mitochondrion [10, 51, 52]. Finally, the autophagosome fuses with the lysosome and the mitochondrion is degraded.

In short, the diversity of mitochondrial receptors highlights the existence of compensatory mechanisms which sometimes work in concert. Maintaining mitochondrial function through these receptors is essential for energy metabolism and cell homeostasis.

## Compromised mitophagy in AD patients and animal models

Recognized features of mitochondrial dysfunction in AD, including oxidative stress, free radical accumulation, perturbed energy metabolism, intracellular calcium imbalance, and enzymatic activity reduction, are related to mitochondrial bioenergetics [8, 53, 54]. Mitochondrial dysfunction is a prominent neuronal feature of early AD; it affects glucose use and energy metabolism in pre-AD patients with mild cognitive impairments [55]. This might reflect defects in mitochondrial dynamics, distribution, and bioenergetics, such as irregular distribution (reduced number), damaged morphology (fragmentation, ultrastructural damage), and altered bioenergetics (membrane potential, cytochrome *c* oxidase activity, ATP production), as shown by several studies using in vivo and in vitro AD models [56–60]. In human studies, Bohr et al. have compared mitochondrial morphology in the hippocampus of AD patients with age- and gender-matched healthy individuals and found mitochondrial size reduction and excessive mitochondrial damage in AD samples [11]. Using the fluoro-deoxy-glucose positron emission tomography imaging technique, researchers have reported that the brains of AD patients have a significantly lower glucose utilization rate than those of healthy subjects [55, 61]. Since then, mitochondrial dysfunction has been found to play a critical role in AD pathogenesis. In mice, amyloid precursor protein (APP) overexpression in neurons began to cause mitochondrial dysfunctions, such as mitochondrial membrane potential reduction and ATP level decrease, at 3 months. Mitochondrial dysfunction is also associated with higher reactive oxygen species (ROS) levels, altered B-cell lymphoma-extra large (Bcl-xL)/ Bcl-2-associated X protein (Bax) ratio, and reduced cytochrome *c* oxidase complex IV (COX IV) activity [62]. Resende et al. documented that the activity of the antioxidant enzymes glutathione peroxidase and superoxide dismutase (SOD) increases during the Aβ oligomerization period [62]. In other words, oxidative stress occurs early in AD development, before Aβ plaques and neurofibrillary tangles are observed [62]. Mitophagy, as a clearance process for damaged mitochondria and one of the mitochondrial quality control pathways, attracts a lot of attention in AD studies. Lots of clinical and animal model studies have revealed the importance of compromised mitophagy in AD.

To clear off impaired mitochondria, autophagosomes containing them must fuse with lysosomes to form autolysosomes. Moreover, impaired autophagy is one of the hallmarks of AD [63]. Thus, accumulation of non-degraded dysfunctional mitochondria in cells may result from the combined effects of lysosomal dysfunction and mitochondrial transport disorder [64]. AD patients display accumulation of autophagic vacuoles in both neuronal and peripheral cells [65, 66], and their pyramidal neurons contain high levels of mitochondria-containing autophagic vesicles [67, 68]. Another cause of damaged mitophagy in AD may be the obstacle in autophagosome-lysosome fusion [69–71].

Studies in AD patients and animal models have reported changes in mitophagy-related genes. For example, in 2017, Sorrentino et al. reported the relationship between mitochondrial protein homeostasis and AD pathogenesis [72]. They performed gene set enrichment analysis on data from the GeneNetwork database in the prefrontal cortex, primary visual cortex, and the entire brain of 388 AD patients and 195 healthy people. They showed that downregulation of the oxidative phosphorylation pathway and dysfunction of the mitochondrial import pathway are both characteristic features in AD patients [72]. Furthermore, AD patients with mild cognitive impairment and mild/moderate AD had higher transcriptional levels of several genes related to mitophagy, such as p62, PARKIN, dynamin 1-like, and beclin 1, compared to people without cognitive impairment [72]. Another study found that 6- and 9-month-old 3-× Tg AD mice have lower mRNA levels of mitophagy-related genes than wild-type C57 mice [72].

However, other researchers have observed different phenomena. Bohr et al. also compared the mitochondrial morphology in hippocampal neurons of AD patients and age- and gender-matched healthy individuals; he observed a reduced mitochondrial size and excessive mitochondrial damage in AD samples [11]. Moreover, some AD patients have low levels of mitophagy proteins such as PINK1, Bcl-2-like protein 13, and BNlP3L/NIX, and the mitophagy initiation proteins p-TANK-binding kinase 1(TBK1) (Ser172)16 and p-unc-51 like autophagy activating kinase 1 (ULK1) (Ser555)15 are inactivated in all human AD samples, indicating a potential relationship between mitophagy deficits and accumulation of dysfunctional mitochondria in the brains of AD patients [11]. These differing results [11, 72] may be due to the different disease stages in the studied AD patients. Mitophagy may be a compensatory change after mitochondrial damage in AD patients. Furthermore, disease development may weaken the compensatory ability. Therefore, changes in the transcription and protein levels of mitophagy-related genes may fluctuate from patient to patient and according to disease stage. As Sorrentino et al. observed, the transcription levels of mitophagy-related genes, such as p62, PARK2, PINK1, and LC3, are lower in 9-month-old than in 6-month-old 3-× Tg AD mice [72]. The development of bioinformatics tools, induced pluripotent stem cells (iPSC), single-cell RNAseq, and other technologies will allow us to better understand the mitophagy changes at different AD stages and their roles in AD pathogenesis.

## The interaction between defective mitophagy and Tau and/or Aβ

Although existing studies have confirmed that AD patients show more mitochondrial dysfunction, including mitophagy disorder, than other populations, the role of abnormal mitophagy in the AD pathological process remains unclear. At present, the exploration of the interaction between mitophagy and two major pathological features of AD—Aβ aggregation and Tau hyperphosphorylation, which result in senile plaques and neurofibrillary tangles, respectively—is still in its infancy.

### Mitophagy and Tau pathology

Human Tau, encoded by the *MAPT* (microtubule-associated protein tau) gene, is mainly distributed in axons and stabilizes microtubules in neurons [73]. The hyperphosphorylation of Tau, the major component of neurofibrillary tangles, reduces its affinity for microtubules, destabilizing the cytoskeleton, causing synaptic dysfunction, and impairing the axonal transport of organelles [74, 75]. Thus, Tau plays a vital role in AD pathogenesis. AD samples have markedly fewer mitochondria in Tau-positive neurons than control tissues do [76], which may be associated with the mitochondrial transportation impairment. Hyperphosphorylated Tau also inhibits kinesin-dependent peroxisome transport, making cells vulnerable to oxidative stress and degenerating affected neurons [77]. Other pathological forms of Tau, such as P301L Tau, also cause mitochondrial dysfunction and, together with reduced nicotinamide adenine dinucleotide-ubiquinone oxidoreductase activity, increase ROS production and impair mitochondrial respiration and ATP synthesis [78]. P301S Tau transgenic mice also exhibit mitochondrial dysfunction and elevated oxidative stress, including increased protein carbonyl levels in the mitochondria and alterations in the activity and levels of mitochondrial enzymes involved in ROS formation [79]. These changes could be associated with glycogen synthase kinase 3 beta (GSK3β) activation [79]. Granulovacuolar degeneration, related to early Tau pathology in AD brains, also contributes to mitophagy impairments [80].

Conversely, increased mitochondrial oxidative stress could modulate Tau phosphorylation both in vitro and in vivo [81–83]. In a chronic oxidative stress model, inhibiting glutathione synthesis in M17 cells increased Tau phosphorylation at GSK3β-specific Ser396/Ser404 (PHF-1) sites. This effect is likely mediated by the increased c-Jun N-terminal kinase and p38 activity and decreased protein phosphatase 2A activity [81]. Furthermore, H_2_O_2_-induced oxidative stress leads to Tau hyperphosphorylation via GSK3β activation [82]. In an in vivo study, mitochondrial SOD2 deficiency exacerbated amyloid burden and increased the level of Ser396-phosphorylated Tau. Meanwhile, treatment of *Sod2-*null mice (which normally die within the first week of life) with a high dose of catalytic antioxidant reduced Tau phosphorylation at Ser396, while a low dose did not [83]. Therefore, cumulative mitochondrial dysfunction may also promote Tau hyperphosphorylation through oxidative stress, further participating in the process of AD.

**Fig. 2:**
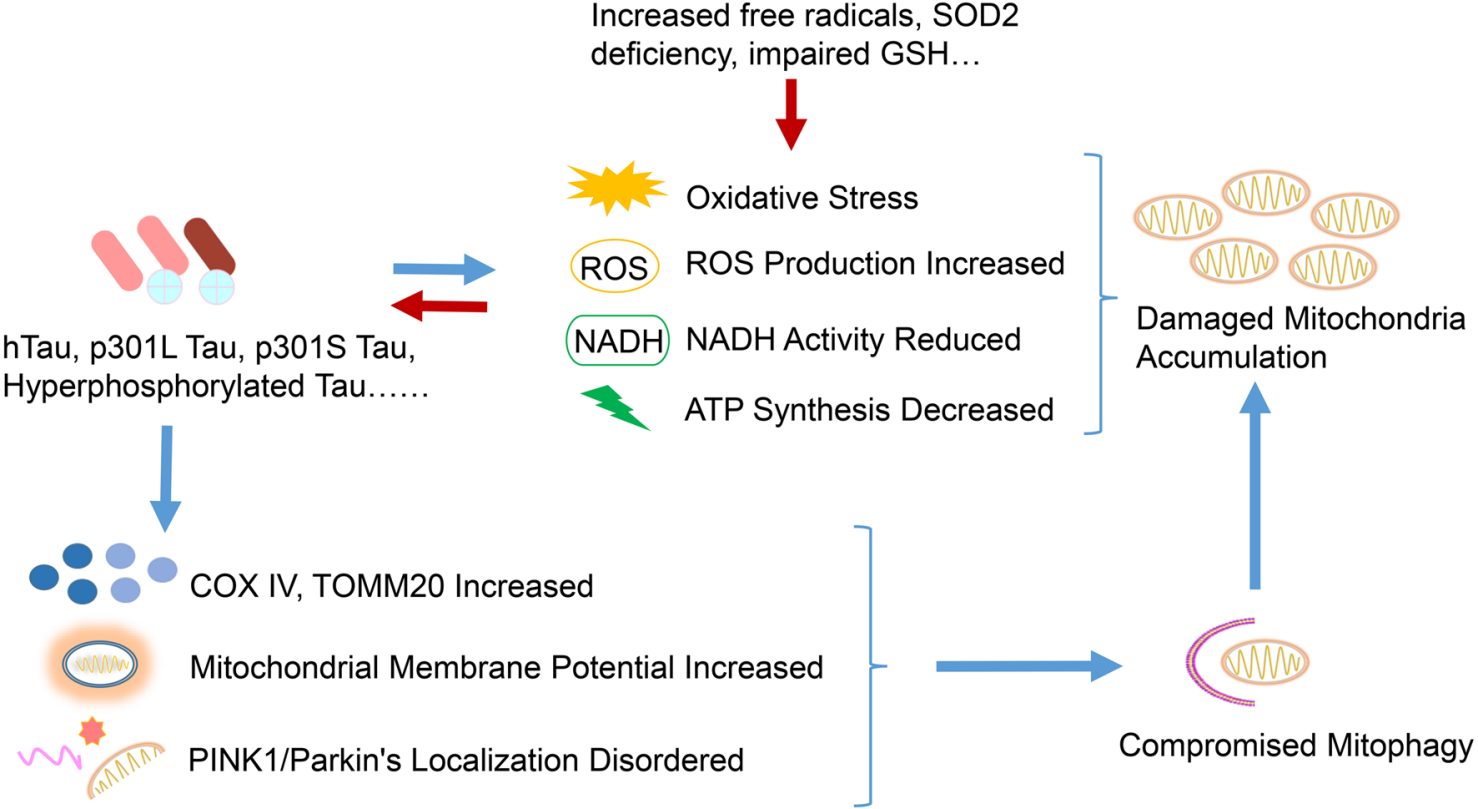
Interactions between mitophagy and Tau pathology. Abnormal Tau, together with increased free radicals, SOD2 deficiency, impaired glutathione synthesis and other assaults, causes mitochondrial damage by increasing oxidative stress and ROS production, as well as inducing NADH activity and ATP synthesis. Meanwhile, these mitochondrial dysfunctions increase pathological Tau levels, which compromise mitophagy by increasing COX IV and TOMM20 levels, mitochondrial membrane potential, and disrupt PINK1/parkin localization, further aggravating mitochondrial damage and mitophagy disorder. Thus, the damaged mitochondria accumulate in neurons

Like normal mitochondrial function, mitophagy is impaired in AD. Yu et al. reported that full-length human Tau (hTau) protein accumulation disrupts mitophagy. They found high levels of COX IV and translocase of outer mitochondrial membrane 20 (TOMM20) proteins and mitochondrial-to-genomic DNA ratio in AD patient brain and hTau transgenic mice. It is noteworthy that these alterations were only found in the brains of AD patients with an increased total Tau level [84]. The accumulated intracellular hTau is directly inserted into the mitochondrial outer membrane and increases the mitochondrial membrane potential, disrupting the localization of PINK1/parkin on the mitochondria and thus mitophagy [84]. Overexpressing hTau in HEK293 cells, primary hippocampal neurons, and C57 mouse brains decreases mitochondrial PINK1 and parkin levels, while upregulating parkin attenuates this decrease [84]. Besides the full-length Tau, pathological Tau also influences mitophagy. Amadoro et al. found a 26–230 amino acid-long Tau protein segment (20–22 kDa) called NH_2_hTau in the cerebrospinal fluid and synaptic mitochondria of AD patients and in animal or cell models [85]. Moreover, this NH_2_hTau is substantially cytotoxic in primary hippocampal neurons and can impair mitochondrial biological properties, either directly by inhibiting ADP/ATP exchange (which depends on ANT-1) or indirectly by affecting mitophagy [86]. The authors then found that excessive NH_2_hTau accumulation under pathological conditions would abnormally recruit parkin and ubiquitin carboxyl-terminal hydrolase isozyme L1 (UCHL-1) to form an NH_2_hTau fragment/parkin/UCHL-1 complex that overdrives mitophagy, resulting in mitophagic neuronal death [86]. However, recent studies showed that different pathological Tau proteins can affect mitophagy differently, and both hTau and frontotemporal dementia mutant Tau (hP301L) inhibit mitophagy in neuroblastomas by reducing parkin transfer to the mitochondria [87]. In the nervous system of *Caenorhabditis elegans*, hTau expression reduces mitophagy, whereas hP301L expression completely inhibits it [87]. These results are not caused by changes in mitochondrial membrane potential or cytoskeleton, but by the impaired recruitment of defective mitochondria by parkin, which inhibits mitophagy [87] (Fig. 2).

These findings add a new dimension to our understanding of Tau toxicity, illustrating the interference and effects of different Tau forms on mitophagy.

### Mitophagy and Aβ toxicity

APP is a transmembrane protein with a large N-terminal extracellular tail and an Aβ domain partly embedded in the plasma membrane [5]. APP can be cleaved by α-, β-, and γ-secretases. The α-secretase cleaves it on the membrane surface to release a non-toxic secretory APP, while β- and γ-secretases sequentially cleave the complete Aβ fragment from the N- and C-terminal ends of APP [88]. Extracellular amyloid plaques consist of abnormally folded 40- or 42-amino acid-long Aβ (Aβ40 and Aβ42). The amyloid hypothesis suggests that AD results from the accumulation of pathological Aβ forms in the brain, driven by an imbalance between Aβ production and Aβ clearance [89]. Substantial evidence suggests that mitochondrial dysfunction and oxidative stress interact to promote Aβ-oriented APP cleavage and accumulation. Mitochondria of 3-month-old London/Swedish APP double mutant transgenic mice with high intracellular but low extracellular Aβ deposits have low mitochondrial membrane potential and ATP levels [62]. This is associated with high ROS levels, altered Bcl-xL/Bax ratio, and reduced COX IV activity [62]. Tg2576 mice, an AD model overexpressing human APP, display increased hydrogen peroxide and decreased cytochrome *c* oxidase activity before Aβ plaques appear, while mutant APP and soluble Aβ impair mitochondrial metabolism by producing hydrogen peroxide and decreasing cytochrome *c* oxidase activity [90]. Moreover, non-glycosylated full-length and C-terminal-truncated APPs accumulate exclusively in the protein import channels of mitochondria of AD patient brains but not in age-matched controls, and the abnormal accumulation of APP across mitochondrial import channels directly correlates with mitochondrial dysfunction [91]. APP C-terminal fragment accumulation leads to mitochondrial structure alterations and failure of basal mitophagy independently of Aβ, both in vivo and in vitro [71]. APP also causes an imbalance of mitochondrial fission/fusion by significantly decreasing dynamin-like protein 1 and OPA1 levels and increasing Fis1 levels, resulting in mitochondrial fragmentation and abnormal distribution [92]. Mitochondrial antioxidant manganese SOD2 protects the aging brain against human APP/Aβ-induced impairments [93]. Prdx3, a key enzyme involved in detoxifying mitochondrial H_2_O_2_, also suppresses NLRP3 inflammasome activation, reduces brain inflammation, and attenuates cognitive impairment of APP/PS1 after paraquat exposure [94]. Thus, mutant APP and Aβ impair mitochondria in the early stage of AD, and inhibiting mitochondrial dysfunction may attenuate Aβ pathology.

In human AD patients and mice overexpressing the Swedish and Indiana mutations, primary cortical neurons have remarkably active parkin-induced mitophagy, a process that is independent of any human intervention on mitochondrial membrane potential [95]. The depolarized mitochondria recruit high levels of parkin, and parkin translocation mainly occurs in somatodendritic regions, which is related to a decrease of anterograde and an increase of retrograde transport of axonal mitochondria [95]. Another study revealed that the primary hippocampal neurons overexpressing mutant APP (also Swedish/Indiana mutations) by transfection express significantly lower mRNA and protein levels of mitophagy-related genes, including PINK1, telomerase reverse transcriptase (TERT), Bcl-2, and BNIP3L than the control group. Meanwhile, the autophagy-related genes *ATG5*, *Beclin 1*, *LC3A*, and *LC3B* are upregulated [96]. The same study also revealed that mitochondrial fission-related genes *Drp1* and mitochondrial fission 1 (*Fis1*) are upregulated, while mitochondrial fusion-related genes *Mfn1*, *Mfn2*, and *Opa1* are downregulated [96]. Another study using Tg2576 mice overexpressing mutant human APP, reported similar results. The authors found that 12-month-old APP mice have lower levels of proteins related to mitophagy (PINK1 and TERT), autophagy (ATG5, LC3BI, and LC3BII), fusion (Mfn1, Mfn2, and Opa1), and biogenesis (peroxisome proliferator-activated receptor-γ co-activator-1α, NRF1, NRF2, and human mitochondrial transcription factor A), and higher levels of mitochondrial fission proteins, including Drp1 and Fis1, than age-matched non-transgenic wild-type mice [97]. Therefore, although APP mutations can induce parkin-mediated mitophagy, the mitophagy may eventually be compromised by the consumption and depletion of mitophagy-related proteins.

In the absence of APP mutations, direct stimulation with Aβ also has a certain effect on mitophagy. Injecting Aβ into the hippocampal CA1 area of rats increased autophagy from the second day, and the LC3II-LC3I/p62 and Atg7/Atg12-5 complex ratios increased and reached a peak on the fifth day [97]. The changes of mitophagy-related proteins PINK1 and parkin were basically consistent with those of autophagy, but both of them decreased back to normal levels when the level of apoptosis marker caspase-3 increased, while mitochondrial fission/fusion increased, and mitochondrial biogenesis significantly decreased [97]. In another report, stimulation of mouse primary cortical neurons with 10 μM Aβ1-42 affected several mitochondria-related processes. It increased mitophagy by upregulating LC3B and PINK1, depolarized mitochondrial membrane potential, induced mitochondrial damage, apoptosis and mitochondrial fission, and increased intracellular ROS levels [98]. Interestingly, in parkin-overexpressing 3-× Tg mice, the parkin-induced mitophagy could clear off intracellular Aβ and restore the levels of processes such as oxidative stress, mitochondrial dysfunction, ATP and GABA production, and neurotransmitter balance, thereby decreasing neuronal death and degeneration [99]. Together, these results suggest that Aβ stimulation could induce mitophagy in a process that could either be a self-regulation strategy or a neuronal protection system.

Thus, it is tempting to conclude that APP mutation or Aβ toxicity plays a regulatory role through mitophagy induction. However, energy disturbance and impaired mitophagy caused by APP mutations and Aβ aggregation weaken the neuronal compensatory mechanisms. These, along with the damaged organelles and toxic protein accumulation in cells and tissues, further aggravate the situation by initiating a vicious cycle causing neuronal damage and death.

### Tau and Aβ cooperatively impair mitophagy

AD is a multifactorial disease with many intermingling pathological changes resulting in disease progression. Several abnormally modified proteins (Aβ, Tau, etc.) accumulate due to impaired autophagy and mitophagy. These abnormal proteins sometimes interact with each other to produce harmful effects. For example, Tau inhibits APP transport into axons and dendrites, causing its accumulation in the soma [84]. Many studies have reported common or synergic influences that Aβ and Tau exert on mitophagy.

The different pathological Tau proteins can work in synergy with or aggravate Aβ-induced mitochondrial impairments. Studies have confirmed that caspase-3 cleaves Tau at Asp421 before neurofibrillary tangles form, promoting the early AD onset [98]. This pathological Tau in the primary neurons can worsen the abnormal mitochondria localization and transport as well as oxidative stress caused by low-concentration Aβ [99]. Further evidence also revealed that overexpressing pseudo-phosphorylated Tau at the PHF-1 epitope (S396/S404) in mature neurons does not noticeably alter mitochondrial morphology, length, or transport, but treating them with Aβ enhances the Aβ-induced loss of mitochondrial membrane potential and increased SOD levels [100]. Besides pathological Tau, full-length Tau promotes similar changes in mitochondrial membrane potential and mitochondrial dysfunction following Aβ treatment. This effect is independent of cytoplasmic calcium concentration [101].

Meanwhile, although APP and Tau overexpression increases autophagy flux following carbonyl cyanide *m*-chlorophenyl hydrazine treatment, the recruitment of parkin and PINK1 by mitochondria is reduced, resulting in dysfunctional mitochondria accumulation [102]. Remarkably, accumulation of depolarized mitochondria requires combined Tau and APP. Thus, in this cell model, Tau and APP overexpression simply reproduces the impaired mitophagy found in sporadic AD.

Using a quantitative mass-tag labeling proteomic technique (with the iTRAQ reagent) and mass spectrometry, researchers observed markedly deregulated levels of numerous proteins in 3-× Tg mice. One-third of them are mitochondrial proteins, mainly related to oxidative phosphorylation complexes I and IV. Interestingly, the activity deregulation of complex IV is associated with Aβ, whereas complex I deregulation is associated with Tau [103]. Moreover, the same study also showed that 8-month-old 3-× Tg mice have obvious mitochondrial impairments (evidenced by a decrease in mitochondrial membrane potential). These impairments worsen in 12-month-old mice (reflected in tricarboxylic acid cycle, oxidative phosphorylation, and ATP synthesis impairments, and increased ROS generation) [103]. Thus, the age-dependent dysfunction of AD animals can also be seen as the vicious circle caused by mitochondrial dysfunction. By comparing the three AD mouse models (APP, APP/PS1, and 3-× Tg mice), some researchers found that APP/Aβ and phosphorylated Tau interact with VDAC1 on the mitochondrial membrane, blocking pores on neuronal mitochondria [104]. This may be one of the reasons why Tau and Aβ jointly aggravate mitochondrial injury [104].

**Fig. 3:**
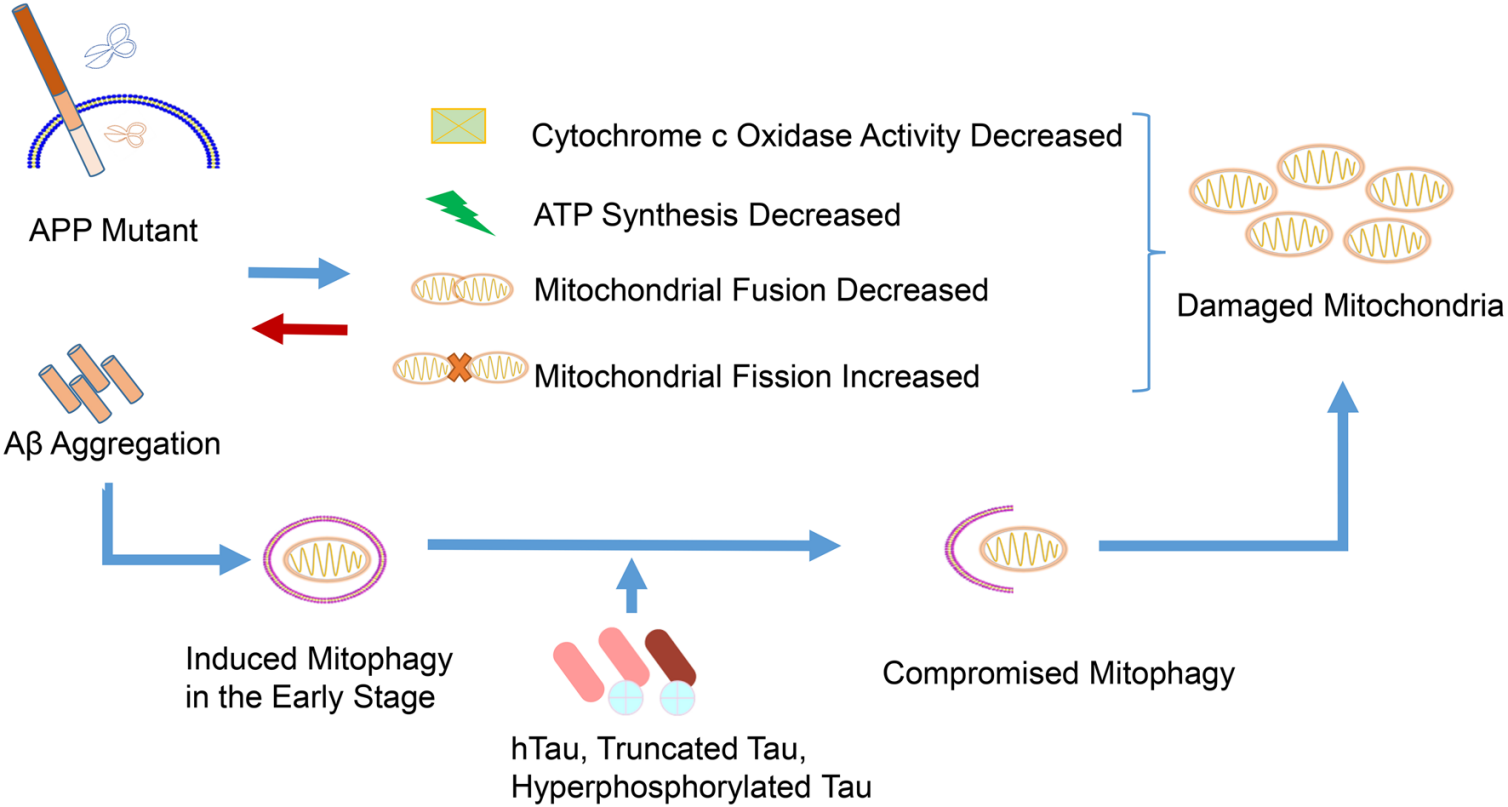
Interactions between mitophagy and APP/Aβ and Tau toxicity. The presence of AD-favoring APP mutations increases Aβ levels. This impairs mitochondrial functions by decreasing the expression of autophagy- and mitophagy-related proteins and genes, decreasing cytochrome *c* oxidase and ATP levels, decreasing mitochondrial fusion, and increasing fission. All these factors damage mitochondria, as well as promoting Aβ-oriented APP cleavage and accumulation. At the early stages of AD, mitophagy can alleviate this damage. However, as AD progresses, Aβ and abnormal Tau accumulate, eventually compromising mitophagy. As a result, the damaged mitochondria cannot be cleared and therefore accumulate

Therefore, mitochondrial dysfunction appears in the early stages of AD, but the subsequent Aβ aggregation and tauopathy, individually or synergistically, worsen the most crucial energy-producing organelle. This triggers the accumulation of damaged and dysfunctional mitochondria, which is closely related to mitophagy impairment (Fig. 3). However, there is still a long way to go to understand the molecular mechanisms of mitophagy impairments in AD, and more specific cellular and molecular mechanisms and key proteins are yet to be discovered.

## The potential of targeting mitophagy to treat AD

The findings discussed above show that mitophagy-related proteins are obviously affected in AD patients and AD animal models. Some reports suggest complex interactions between mitophagy and AD pathology processes such as Aβ aggregation and hyperphosphorylated Tau accumulation. Thus, therapeutic interventions aiming to restore mitophagy functions are promising treatment strategies for AD.

Parkin overexpression restores mitochondrial parkin and LC3-II levels in Aβ-treated cells by repairing damaged mitophagy and inhibiting Aβ-induced mitochondrial dysfunction [105]. In 3 × Tg mice, parkin overexpression also stimulates the beclin-dependent molecular cascade of autophagy and facilitates the clearance of vesicles containing debris and defective mitochondria [106]. Moreover, parkin protects against oxidative stress and mitochondrial dysfunction, removes intracellular Aβ, and restores energy metabolism and neurotransmitter balance [106]. In addition, PINK1 overexpression in APP/PS1 mice has the same effects. PINK1 overexpression enhances autophagy signaling by activating autophagy receptors OPTN and NDP52, thereby promoting clearance of damaged mitochondria, reducing oxidative stress, and alleviating synaptic damage and cognitive decline in APP/PS1 mice [107]. Studies on the role of non-transcribed microRNAs in mitophagy reveal that inhibiting miR-204 expression can efficiently inhibit AD progression by improving mitophagy [108].

Pharmacologic induction of mitophagy, such as with the use of urolithin A, actinonin, or nicotinamide mononucleotide, has beneficial effects on Aβ and Tau pathologies and improves cognitive dysfunction in various AD models, including iPSC-derived neurons, transgenic nematodes, and AD mouse models [11]. These three potent mitophagy inducers trigger neuronal mitophagy through several key mitophagy genes, including dct-1, pdr-1, and pink-1, restore mitochondrial morphology and size in the hippocampus of AD mice, and decrease mitochondrial ROS levels [11]. Cholesterol can also affect PINK1-parkin-mediated mitophagy via OPTN. Indeed, decreasing brain cholesterol together with administration of autophagy inducers is a potential treatment for AD [109]. Recent studies also revealed that melatonin, honokiol, and UMI-77 could alleviate cognitive impairment by promoting mitophagy in AD animal models [110–112].

**Fig. 4:**
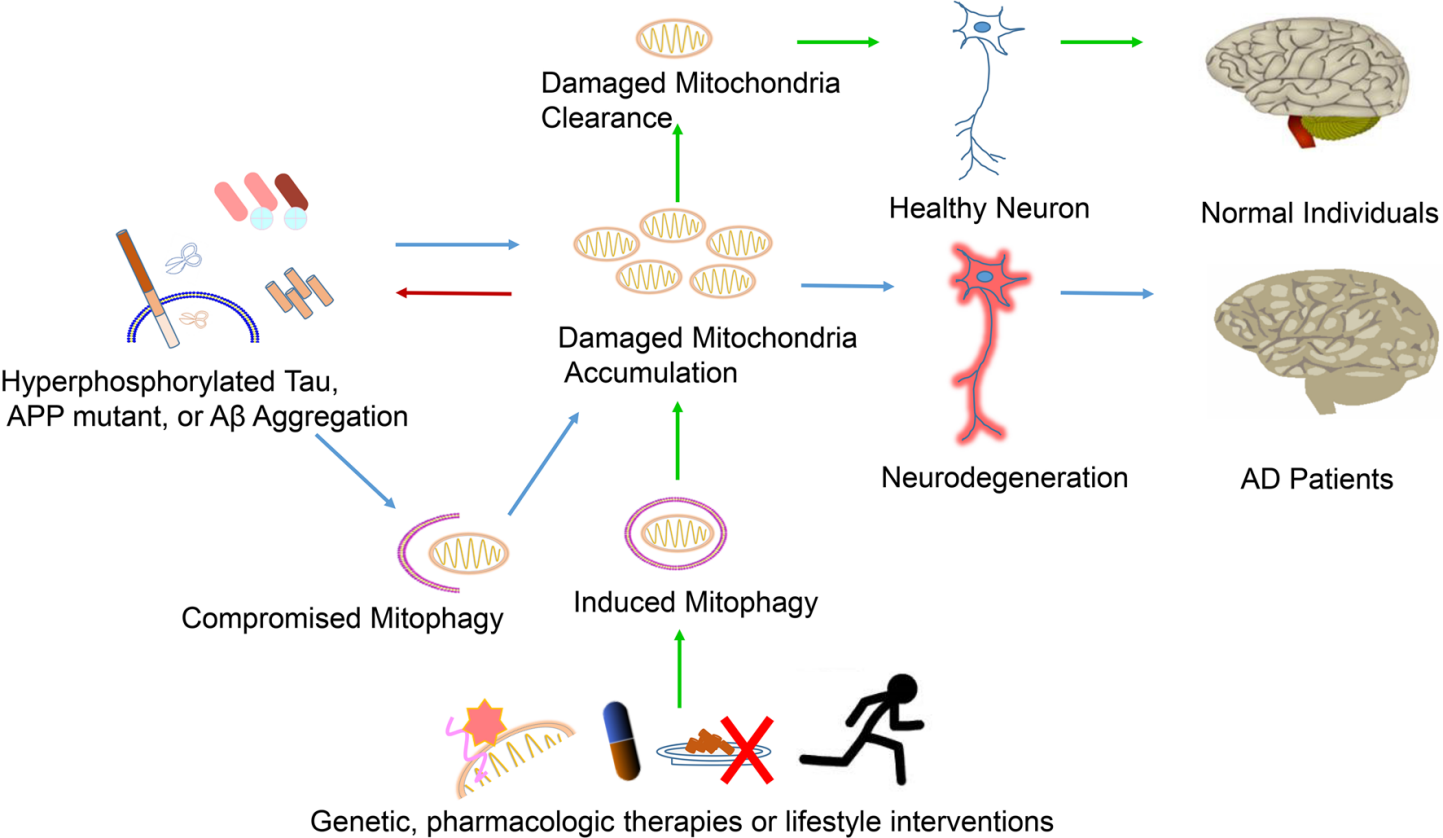
Genetic and pharmacologic therapies or lifestyle interventions targeting mitophagy are potential strategies to prevent and treat AD. Abnormal Tau, APP mutant or Aβ leads to mitochondrial impairments, which in turn aggravate Tau and Aβ pathologies. These pathological changes further compromise mitophagy, which decreases the clearance of damaged mitochondria, forming a vicious cycle. This process triggers the accumulation of damaged mitochondria, consequently decreasing the energy supply to neurons which, along with other processes, leads to neurodegeneration and brain atrophy. However, genetic and pharmacologic therapies or lifestyle interventions, such as exercise and fasting, could induce mitophagy and clear the damaged mitochondria. Thus, these strategies have great potential in preventing and treating AD

In animal models, lifestyle changes can also improve mitochondrial health and promote mitophagy. Caloric restriction, intermittent fasting, and exercise are bioenergetic challenges that promote neuroplasticity (synapse formation, hippocampal neurogenesis) and learning and memory abilities, and improve resistance to neuronal stress [113, 114]. Caloric restriction, intermittent fasting, and vigorous exercise can reduce mitochondrial oxidative stress, stimulate mitochondrial biogenesis, enhance autophagy, and promote neuroplasticity, resulting in synaptic formation, hippocampal neurogenesis, and learning and memory improvements [114]. Fasting for 24–48 h increases the autophagosome levels in the cerebral cortical neurons of GFP-LC3 transgenic mice [115]. Additionally, prolonged fasting increases the circulating orexigenic hormone ghrelin, providing neuroprotective effects via activation of AMPK (AMP-activated protein kinase), enhancing mitophagy, and ultimately enhancing mitochondrial bioenergetics [116]. Exercise increases mitophagy by markedly decreasing P62 and PINK1 levels and increasing LC3II and parkin levels in the hippocampus of mice [117]. Finally, exercise reduces the Aβ plaque area and mitochondrial Aβ peptide levels, and increases the levels of synaptic markers synuclein and GAP43 (growth- and plasticity-associated protein-43) in APP/PS1 transgenic mice. It also reverses mitochondrial dysfunction, thereby restoring the ability of learning and memory [117]. Exercise training also prevents cognitive decline in aged rats by promoting mitochondrial biogenesis, increasing mitochondrial fusion and fission, and activating autophagy/mitophagy in aged hippocampal neurons [118] (Fig. 4).

Overall, these results suggest that modulating mitophagy is an effective strategy for AD prevention and treatment, but further studies are needed before its translation into clinical use.

## Conclusion

Many studies have proved that accumulation of dysfunctional mitochondria is a common feature in damaged neurons in AD patients and animal models. As neurons are the most energy-consuming cells of the body, the increased energy demand and decreased supply resulting from impaired mitochondrial function, combined with the increased ROS production and decreased clearance, inevitably lead to neuronal death and cognitive impairments. Mitophagy is an indispensable process for clearance of dysfunctional mitochondria. It regulates mitochondria number and maintains the metabolism. Mitophagy impairment occurs in the early stages of AD and negatively affects a series of biological processes, resulting in Aβ aggregation and Tau hyperphosphorylation. In turn, these pathological changes further compromise mitophagy in a vicious cycle, leading to neurodegeneration and neuronal death. Many studies have shown that stimulating mitophagy, either through lifestyle intervention, gene regulation, or by pharmacological induction, can alleviate AD-like pathological changes as well as learning and cognitive dysfunction in cellular and animal models. However, there are still many puzzles in this field. Are the risk factors of AD, e.g. gender and APOE4, related to compromised mitophagy? And how? What is the time point to intervene mitophagy? Therefore, further studies are needed to elucidate the molecular and cellular mechanisms of mitophagy, understand its specific role in AD occurrence and development, and find therapeutic targets of AD.

## Data Availability

Not applicable.

## Abbreviations

AD: Alzheimer’s disease
Aβ: Amyloid beta
PINK1: PUTATIVE kinase 1
TOM: Translocase of the outer membrane
TIM: Translocase of the inner-membrane
VDAC: Voltage-dependent anion-selective channel protein
DRP1: Dynamin-1-like protein
OPTN: Optineurin
NDP52: Calcium binding and coiled-coil domain 2
LC3: Microtubule-associated protein 1A/1B-light chain 3
LIR: LC3-interacting region
FUNDC1: FUN14 domain-containing protein 1
OPA1: OPTIC atrophy 1
NIX: NIP3-like protein X
BNIP3: Bcl-2 interacting protein 3
BCL2: B cell lymphoma 2
FKBP: FK506‐binding protein
Atg8: Autophagy-related protein 8
GABARAP: Gamma-aminobutyric acid receptor-associated protein
APP: Amyloid precursor protein
ROS: Reactive oxygen species
Bcl-xL: B-cell lymphoma-extra large
Bax: Bcl-2-associated X protein
COX IV: Cytochrome c oxidase complex IV
SOD: Superoxide dismutase
iPSC: Induced pluripotent stem cells
GSK3β: GLYCOGEN synthase kinase 3 beta
TOMM20: Translocase of outer mitochondrial membrane 20
UCHL-1: Ubiquitin carboxyl-terminal hydrolase isozyme L1
TERT: Telomerase reverse transcriptase
Fis1: Mitochondrial fission 1
Mfn1: Mitofusin-1
Mfn2: Mitofusin-1
